# Flow imaging microscopy as a novel tool for high-throughput evaluation of elastin-like polymer coacervates

**DOI:** 10.1371/journal.pone.0216406

**Published:** 2019-05-09

**Authors:** Laura Marvin, Wynter Paiva, Nicole Gill, Marissa A. Morales, Jeffrey Mark Halpern, James Vesenka, Eva Rose M. Balog

**Affiliations:** 1 Department of Chemistry and Physics, University of New England, Biddeford, Maine, United States of America; 2 Fluid Imaging Technologies, Inc., Scarborough, Maine, United States of America; 3 Department of Chemical Engineering, University of New Hampshire, Durham, New Hampshire, United States of America; Aarhus University, DENMARK

## Abstract

Biological and bioinspired polymer microparticles have broad biomedical and industrial applications, including drug delivery, tissue engineering, surface modification, environmental remediation, imaging, and sensing. Full realization of the potential of biopolymer microparticles will require methods for rigorous characterization of particle sizes, morphologies, and dynamics, so that researchers may correlate particle characteristics with synthesis methods and desired functions. Toward this end, we evaluated biopolymer microparticles using flow imaging microscopy. This technology is widely used in the biopharmaceutical industry but is not yet well-known among the materials community. Our polymer, a genetically engineered elastin-like polypeptide (ELP), self-assembles into micron-scale coacervates. We performed flow imaging of ELP coacervates using two different instruments, one with a lower size limit of approximately 2 microns, the other with a lower size limit of approximately 300 nanometers. We validated flow imaging results by comparison with dynamic light scattering and atomic force microscopy analyses. We explored the effects of various solvent conditions on ELP coacervate size, morphology, and behavior, such as the dispersion of single particles versus aggregates. We found that flow imaging is a superior tool for rapid and thorough particle analysis of ELP coacervates in solution. We anticipate that researchers studying many types of microscale protein or polymer assemblies will be interested in flow imaging as a tool for quantitative, solution-based characterization.

## Introduction

Elastin-like polymers (ELPs) are a promising class of protein-based polymers whose therapeutic and materials applications have been widely discussed [1–9]. Composed of repetitive amino acid motifs derived from the vertebrate extracellular matrix protein elastin, ELPs are capable of thermodynamic inverse phase transition to form polymer-rich microparticles called coacervates [10]. The conditions required to trigger coacervation are dependent on ELP composition and are therefore highly tunable [11–13]. Coacervation can also be triggered isothermally by any change to the overall polarity of either the polymer or the solvent, e.g. by increasing the ionic strength of the solution [14,15]. Beyond spherical coacervate droplets, ELPs have also been programmed to form diverse architectures, including micelles, hollow vesicles, cylindrical micelles, and polyhedral virus-like particles [16–20]. Thanks to their stimuli-responsive self-assembly behavior, there has been particular enthusiasm for the potential of ELPs in drug delivery and controlled release applications. For example, ELP coacervates may serve as depots for controlled multivalent molecular display or release of payloads such as drugs, imaging agents, or biosignaling cues [21,22].

One of the simplest methods of studying ELP coacervation is by measuring solution turbidity as a function of temperature with a UV-Visible spectrophotometer; the temperature at which a sharp increase in turbidity is observed is designated the transition temperature (*T*_t_). Dynamic light scattering (DLS) provides much more information about the size distribution of ELPs. Superposition of turbidity and DLS results shows that the sharp increase in turbidity corresponds to formation of micron-scale aggregates, even in samples that form intermediate submicron-scale structures before subsequent bulk aggregation [23]. There are variants of light scattering that can measure rotational diffusion as well as translational diffusion, thereby providing information about particle elongation, but particle geometry is still constructed indirectly from diffusion data [24]. More direct imaging methods such as optical, electron, and scanning probe microscopy are also frequently reported for ELP assemblies; often two or more techniques are used in combination to control against procedural artifacts [25–28]. While microscopy provides both size and shape information, it also requires a large number of particle images for meaningful particle analysis, which can be time-consuming and subjective on conventional instrumentation. For example, AFM characterization is useful for surface-bound structures but cannot reliably provide relevant information about the features of free particles in solution. Additionally, the maximum AFM scan size is limited to 10–100 μm, depending on the piezo scanner’s lateral scanning range, making it difficult to achieve statistically significant numbers of micron-scale particles.

Flow imaging microscopy is increasingly used in the biopharmaceutical industry as a tool for characterizing aggregates and other undesirable sub-visible particles in protein therapeutics [29–32]. In flow imaging microscopy, a fluid sample is pumped through a flow cell that is oriented perpendicularly to a high-magnification optical system [33]. A camera automatically captures images of particles as they move through its field of view. Instrument-specific software can then be used to process, filter, sort, and export image data based on dozens of different measurements including size, shape, symmetry, roughness, intensity, and color. Thousands of individual images are captured within minutes using an automated algorithm, providing statistical power and reducing subjectivity.

Depending on the nature of the materials and the investigation, flow imaging microscopy could complement—or replace with improvement—all of the above techniques. However, despite long-standing and substantial interest in polymer microparticles for therapeutics [34–37], the potential of flow imaging microscopy for these materials has not yet been explored. In this study, we used ELPs as a prototype to investigate the application of flow imaging microscopy to microscale polymer biomaterials.

## Materials and methods

### Protein expression and purification

DNA consisting of the ELP gene (“I40”) coding sequence flanked by BsshII and NheI restriction sites was synthesized by GenScript (Piscataway, NJ, USA). Because ELP sequences can be highly repetitive, codon usage was randomized to prevent recombination. The gene was subcloned into the expression vector POE-W via standard restriction digest and T4 ligation. The POE-W vector tags expressed proteins with a pelB leader signal peptide that directs expressed proteins to the periplasm and is removed after secretion. This vector also provides a single Trp residue tag at the C-terminus to allow protein detection using absorbance at 280 nm. Chemically competent BL21(DE3) *E*. *coli* (New England Biolabs, Ipswich, MA, USA) were transformed with the I40 POE-W plasmid and plated on 2×YT + agar + carbenicillin solid medium. Fresh transformants were picked with a sterile loop and used to inoculate 15 mL starter cultures of 2×YT or SuperBroth + carbenicillin. Starter cultures were grown at 37 °C shaking at 200 rpm until visibly cloudy, typically 2–4 h. The full volume of culture was then used to inoculate 1 L of freshly autoclaved 2×YT or SuperBroth + carbenicillin. Liter cultures were returned to shaking at 37 °C for 24 h following inoculation. The leaky T7 promoter resulted in high levels of recombinant protein production without induction. Cells were harvested by centrifugation (4200*g*, 4 °C, 20 min) and either processed for protein purification immediately or stored at -80 °C. Following periplasmic extraction, I40 was purified using an Inverse Transition Cycling protocol adapted from Hassouneh et al [38]. Following purification, I40 was dialyzed into deionized water, lyophilized, and stored at -20 °C. Our full protocol for purification of ELPs from periplasmic expression systems is available on protocols.io (https://dx.doi.org/10.17504/protocols.io.vfce3iw). Full gene and protein sequence information is provided in the Supporting information (S1 Table).

### Dynamic light scattering

Dynamic light scattering measurements were performed on a Malvern Zetasizer Nano ZS90 DLS at an angle of 175°. To prepare samples, lyophilized I40 was dissolved on ice for at least 10 min using pre-chilled, sterile-filtered deionized water to a final concentration of 12.5 μM. Measurements were made in a clean 1 cm polystyrene cell. Three acquisitions were performed at each temperature, with each acquisition taking 2–3 min. The temperature of the sample was increased from 10–30 °C in increments of 2 °C with a 3 min equilibration at each temperature before acquisition. Peak sizes were assigned based on intensity analysis while relative proportions of different peaks were assigned based on volume distribution analysis.

### Atomic force microscopy

Atomic force microscopy was performed using an Asylum Cypher ES scanning probe microscope (Asylum Research—Oxford Instruments). Amplitude modulated (tapping) mode imaging was performed in water using a BudgetSensors SHR150 probe driven with blueDrive photothermal excitation at a frequency of ~58 kHz. Images were collected as 1024 pixel x 1024 pixel data sets at a scan rate of 0.30 Hz and a scan size of 20.00 μm. To prepare samples, lyophilized I40 was dissolved on ice for at least 10 min in pre-chilled sterile-filtered deionized water to a concentration of 0.3 mg/mL. A small droplet (~20 μL) of I40 solution was placed on Parafilm at room temperature for several minutes to allow coacervation to occur. A freshly cleaved mica substrate was inverted over the droplet and gently touched to the surface of the droplet. The substrate was washed with several 1 mL rinses of room temperature sterile filtered deionized water before imaging. Images were flattened, XY planefit, and had scanner error lines removed.

### Flow imaging microscopy

Flow imaging microscopy was performed on two different instruments: a FlowCam VS and a FlowCam Nano (Fluid Imaging Technologies, Inc.). Measurements were performed using a 50 μm × 1000 μm flow cell and either a 20X (VS) or 40X oil-immersion (Nano) objective. The flow cell was cleaned before each sample run by bath sonication for 3 min. Once connected to the FlowCam, 0.5 mL of water was aspirated three times to rinse any remaining debris. If debris was still visible in the flow cell, the sonication and rinsing procedure was repeated using isopropyl alcohol in place of water. After a final aspiration with water, the FlowCam was focused using 25 μm (VS) or 2 μm (Nano) focus beads (Fluid Imaging Technologies, Inc.). To prepare samples, lyophilized I40 was dissolved on ice for at least 10 min in pre-chilled sterile-filtered deionized water or buffer to a concentration of 1.4 mg/mL, typically in a volume of approximately 1 mL, then allowed to warm passively to room temperature. When 100 μL of this I40 solution was added to 500 μL of water in the pipette tip on the FlowCam sample holder, the final concentration of the sample during analysis was 0.23 mg/mL. For VS measurements, we chose a minimum ESD size acquisition filter of 2 μm and a dark threshold particle segmentation setting of 20. A sample volume of 0.5 mL was analyzed with a flow rate of 0.030 mL/min and a frame rate of 20 frames per second, resulting in approximately one particle per image. For Nano measurements, no minimum size filter was used. Particle segmentation was performed using a dark threshold value of 10 and a light threshold of 25. A sample volume of 0.1 mL was analyzed with a flow rate of 0.020 mL/min and a frame rate of 22 fps, resulting in approximately 50 particles per image. Data were recorded and processed using the built-in FlowCam VisualSpreadsheet program. Any images of focus beads in sample runs were manually selected and used to build a statistical filter (“like selected particles”) so they could be removed before further image analysis. Additionally, an edge gradient filter was applied (minimum value of 40 for VS, 100 for Nano) to ensure only in-focus particles were analyzed. Processed data were exported and graphics were created in Origin 8 (OriginLab). Our full protocol for FlowCam VS analysis of ELPs is available on protocols.io (https://dx.doi.org/10.17504/protocols.io.vg9e3z6).

## Results and discussion

### Synthesis and characterization of the ELP I40

**Fig 1:**
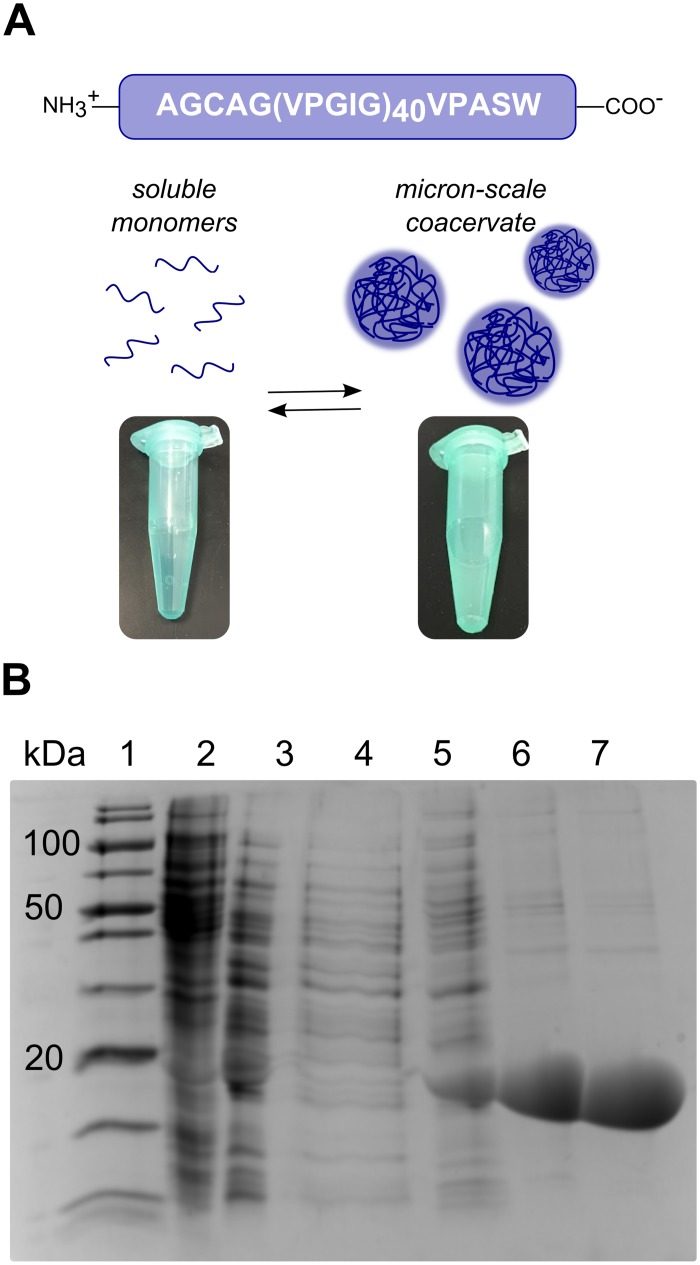
Phase transition of I40 allows purification by temperature cycling. (A) Schematic of I40 ELP and its coacervation behavior. (B) SDS-PAGE analysis of I40 purification. Lane 1: molecular weight marker. Lane 2: first periplasmic extraction fraction. Lane 3: second periplasmic extraction fraction. Lane 4: supernatant following first “hot spin” in 3 M NaCl. Lanes 5–7: “cold spin” supernatants following three subsequent rounds of inverse transition cycling. The uncropped, unlabeled version of this image is provided in the Supporting information (S1 Fig).

We produced the ELP referred to as I40 via recombinant expression in *E*. *coli* and purified the protein using inverse temperature cycling according to the method of Hassouneh et al.[38]. I40 consists of 40 repeats of the motif Val-Pro-Gly-Ile-Gly. A Cys residue near the N-terminus of I40 allows dimerization through disulfide bond formation under non-reducing conditions (sequence provided in Fig 1A and S1 Table). The I40 construct was chosen for convenience, as it expresses well (typical yield ~30 mg/L), purifies easily, and forms coacervates at ambient temperatures. Representative SDS-PAGE analysis of I40 purification is shown in Fig 1B, where I40 runs near its expected size of 17.9 kDa. The reversible coacervation behavior of I40 was observed both during and after the purification process. For all experiments herein, freeze-dried I40 was freshly re-suspended in ice cold water or buffer, then passively brought to ambient temperature, as dry I40 does not easily dissolve in conditions that promote coacervation.

**Fig 2:**
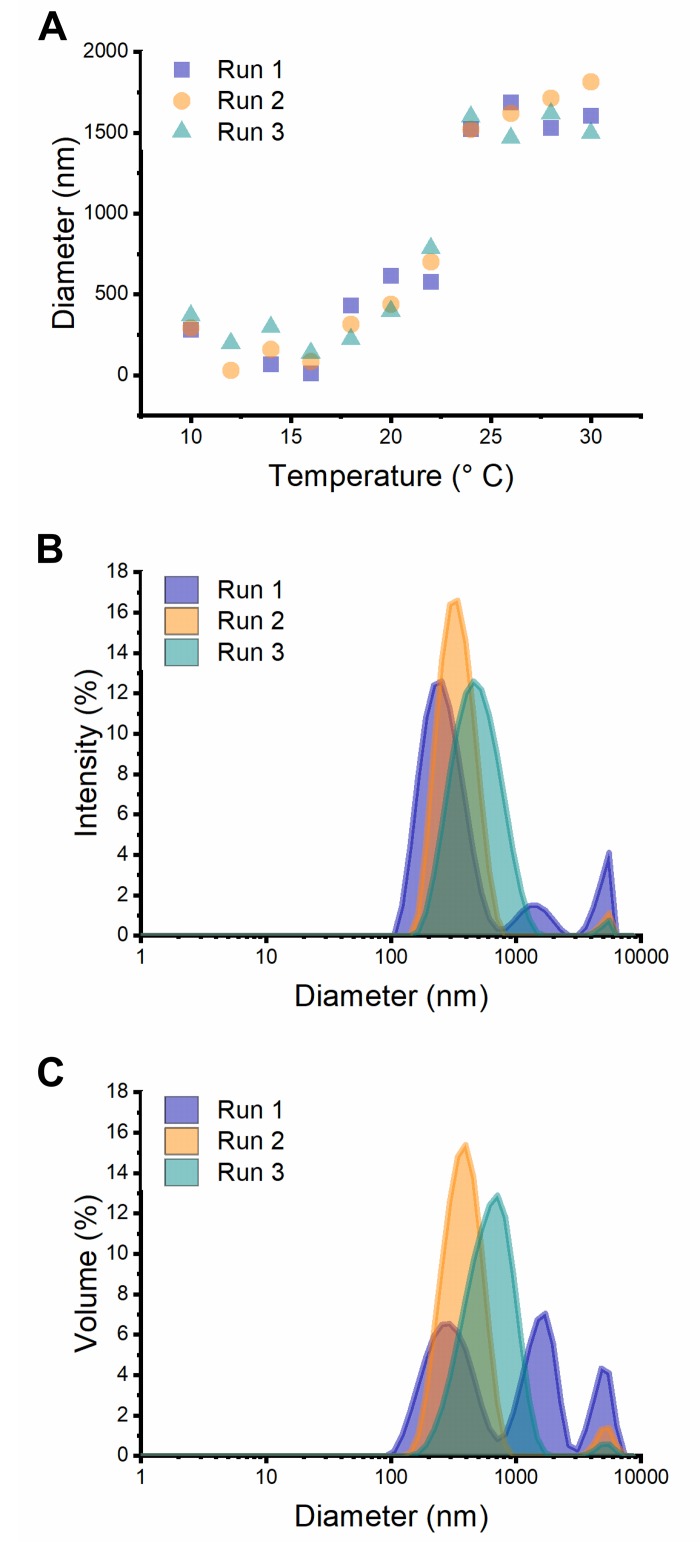
Dynamic light scattering shows temperature-dependent assembly and size distribution of I40 particles. (A) Hydrodynamic diameter of the most relatively abundant species versus temperature. (B) Intensity distribution of particle sizes at 19 °C. (C) Volume distribution of particle sizes at 19 °C.

We analyzed the thermal behavior of I40 in water using DLS (Fig 2). DLS uses scattering intensity to calculate the size of a hypothetical spherical particle with the same diffusional properties as the particles in a sample. For a 0.25 mg/mL (12.5 μM) solution, micron-scale coacervates were the dominant species at ~25 °C and warmer. In samples below 25 °C, we frequently observed high variability in the apparent size distributions across replicate runs (Fig 2B and 2C). For example, at ambient temperatures (~19–24 °C), a multimodal distribution of particles was typically observed, with a dominant peak in the 200–500 nm range and secondary and tertiary peaks with hydrodynamic diameters greater than 1 μm. These data are also consistent with previously reported DLS results for a different ELP (VPGIG_25_) showing a large range of particle sizes in replicate measurements performed close to the *T*_t_ [39]. Therefore, while DLS can be useful for studying ELP temperature transition behavior, our results illustrate the difficulty of obtaining accurate size distribution information for samples that are potentially polymodal, polydisperse, dynamic, self-interacting, and at least partly non-spherical.

**Fig 3:**
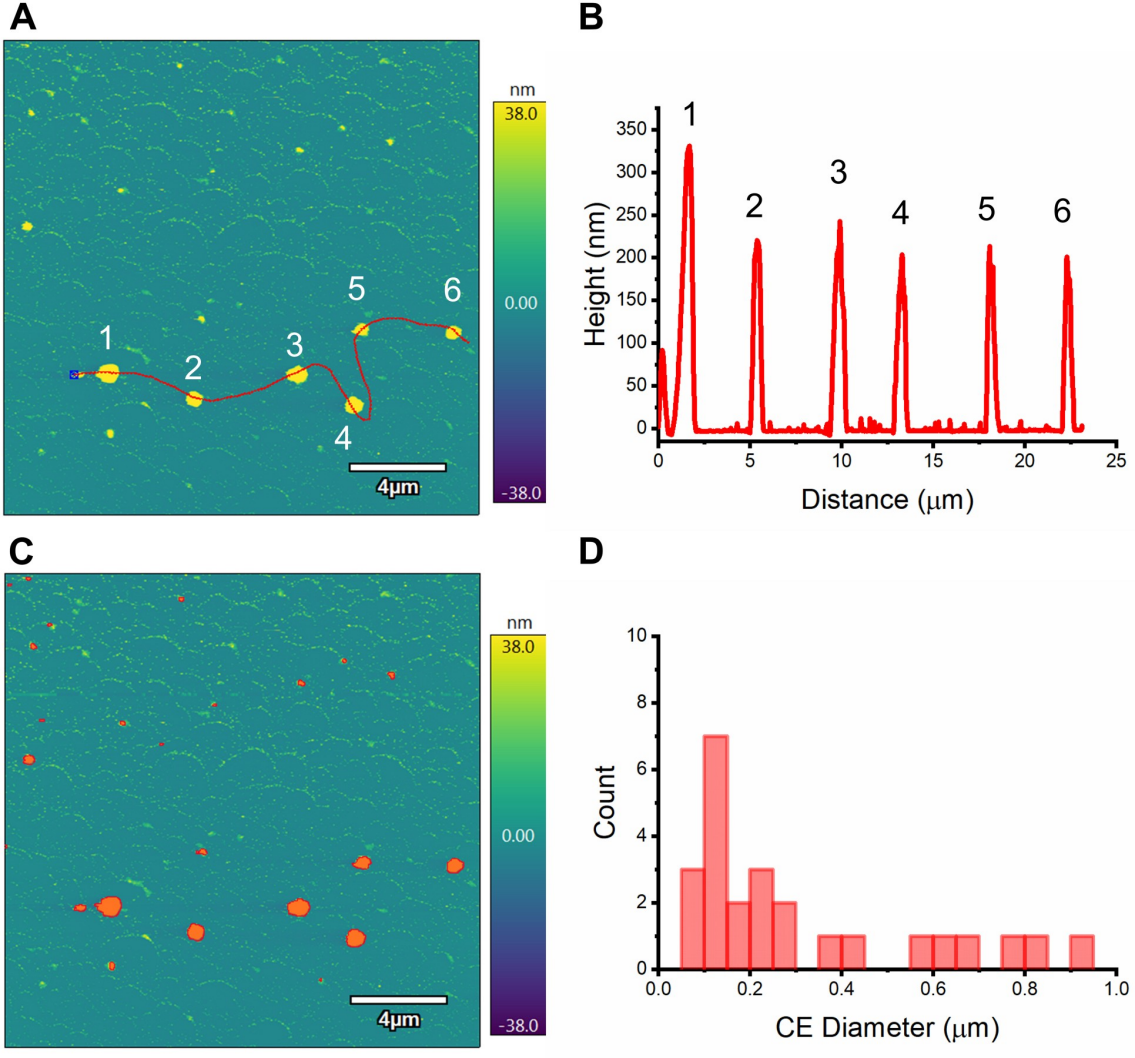
Atomic force microscopy shows size and morphology of I40 particles. (A) AFM height image of I40 assemblies. Red line section corresponds to the height profile in (B). (C) Particle analysis of the same image finds 25 unique particles (red shading). (D) Circle equivalent diameter distribution of particles in (C). The unprocessed version of this image is provided in the Supporting information (S2 Fig).

For comparison, we also performed topographic imaging and particle analysis on I40 assemblies using AFM in liquid. A solution of I40 in water was warmed to room temperature and deposited onto a mica substrate. We observed I40 assemblies with dimensions similar to those observed by DLS (Fig 3). The mean Z-average height (the height value averaged over every pixel) of particles in Fig 3C is 57 nm, while the mean circle equivalent diameter (the diameter of a circle with the same area) for the same population of particles is 318 nm. As the heights of the particles are about one-fifth of their diameters, this indicates very strong adhesion to the mica substrate during the sample preparation process that is not mitigated by imaging in water. Ultimately, at least for this polymer and its assemblies, AFM may be useful for comparing surface-based particle features and behavior under different conditions (e.g., I40 deposited at temperatures above versus below the *T*_t_ shows very different particle populations), but is less useful for quantitative comparison with solution-based techniques such as DLS or flow imaging microscopy.

### Flow imaging assessment of I40 in water

**Fig 4:**
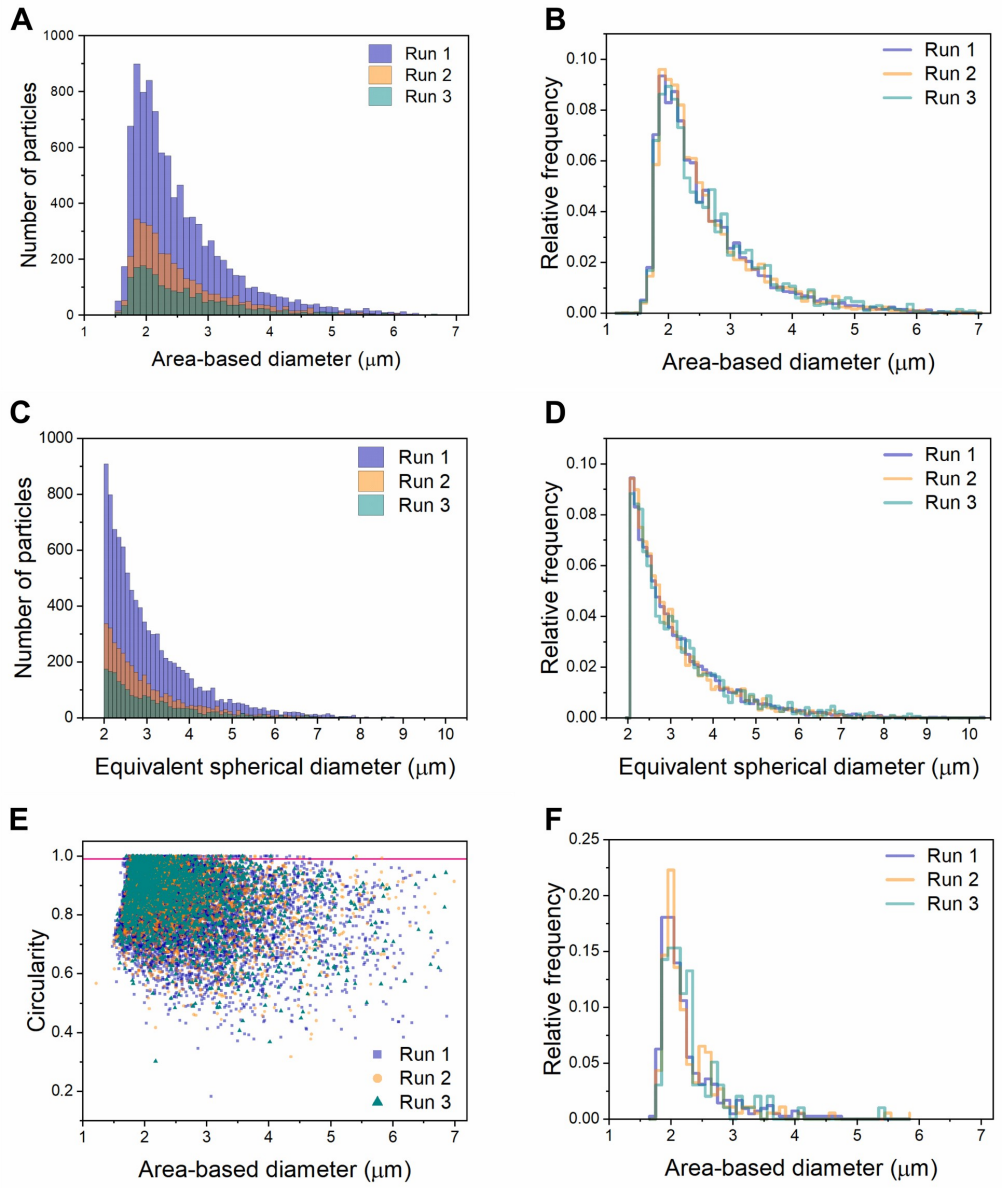
Size and shape characterization of I40 particles in water using the FlowCam VS. Size distribution histograms as defined by area-based diameter (A and B) and equivalent spherical diameter (C and D) for triplicate runs of I40 in water. Panels A and C show the total number of particles, while panels B, D, and F are normalized to show relative frequency. (E) Scatter plot of circularity versus area-based diameter. Pink line represents a circularity value = 0.99. (F) Histogram showing the size distribution of particles with circularity > 0.99, representing single particles.

**Fig 5:**
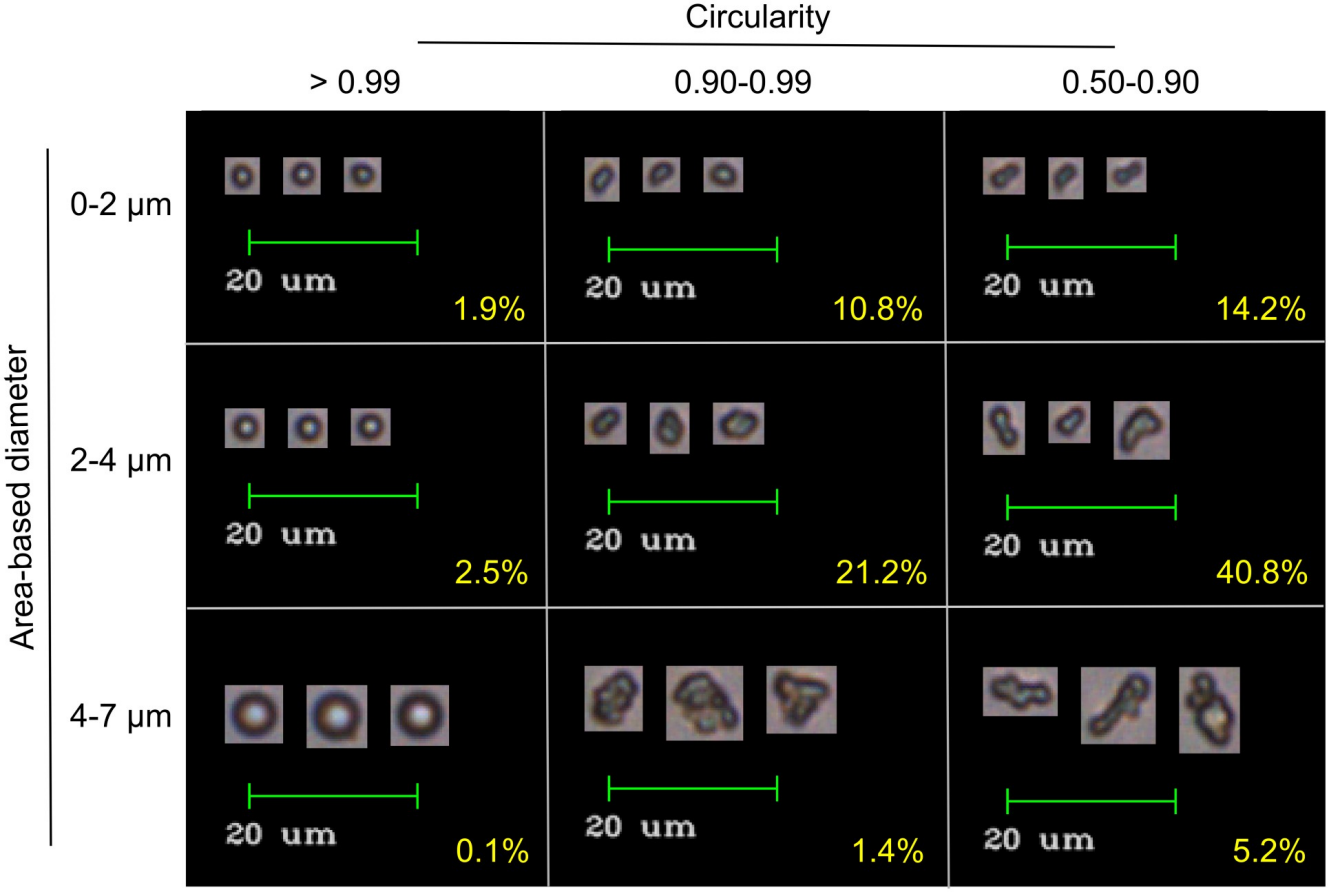
Representative images of I40 particles in water. FlowCam VS images were classified using filters corresponding to the indicated size and circularity bins. Classes were then sorted by edge gradient and the three images with the highest edge gradient from a single run were selected as representative of each class. Percentages of total particles classified into each filter bin from pooled replicate runs are shown in yellow.

Because our DLS results suggested that I40 forms multiple stable or semi-stable species under ambient conditions, we predicted that further characterization of this sample using flow imaging microscopy would provide an illuminating “snapshot” of the coacervation process. Flow imaging generates optical images of particles from which the diameter can be calculated in two ways: (1) area-based diameter (ABD), the diameter of a circle with an area equal to that of the pixels within the particle edge trace, and (2) equivalent-spherical diameter (ESD), the mean of 36 feret diameter measurements of the particle. Fig 4A and 4D show size distributions of the same populations of particles classified by ABD (Fig 4A and 4B) and ESD (Fig 4C and 4D). Three technical replicates of the same sample (collected one after the other from the same tube of re-suspended ELP) are overlaid, showing high run-to-run reproducibility in size distribution despite different total numbers of images per run. Visual inspection of images showed that a large percentage of particles across all sizes are aggregates or fusions of smaller particles. Data were further classified by applying ABD and Hu circularity filters, revealing that particles with circularity greater than 0.99 were reliably single spherical droplets, while particles with lower circularity were often aggregates (Fig 5). Consistent with this, a scatter plot of circularity versus diameter shows clustering in the upper left, indicating smaller, more circular particles (Fig 4E). We found this method of data visualization useful for identifying systematic errors, which we will discuss further below. Fig 4F shows the size distribution of single, spherical coacervates, defined as particles with circularity greater than 0.99 (falling above the pink horizontal line in Fig 4E and shown in Fig 5). Single coacervates show a narrower size distribution falling close to the lower limit of detection for the FlowCam VS.

**Fig 6:**
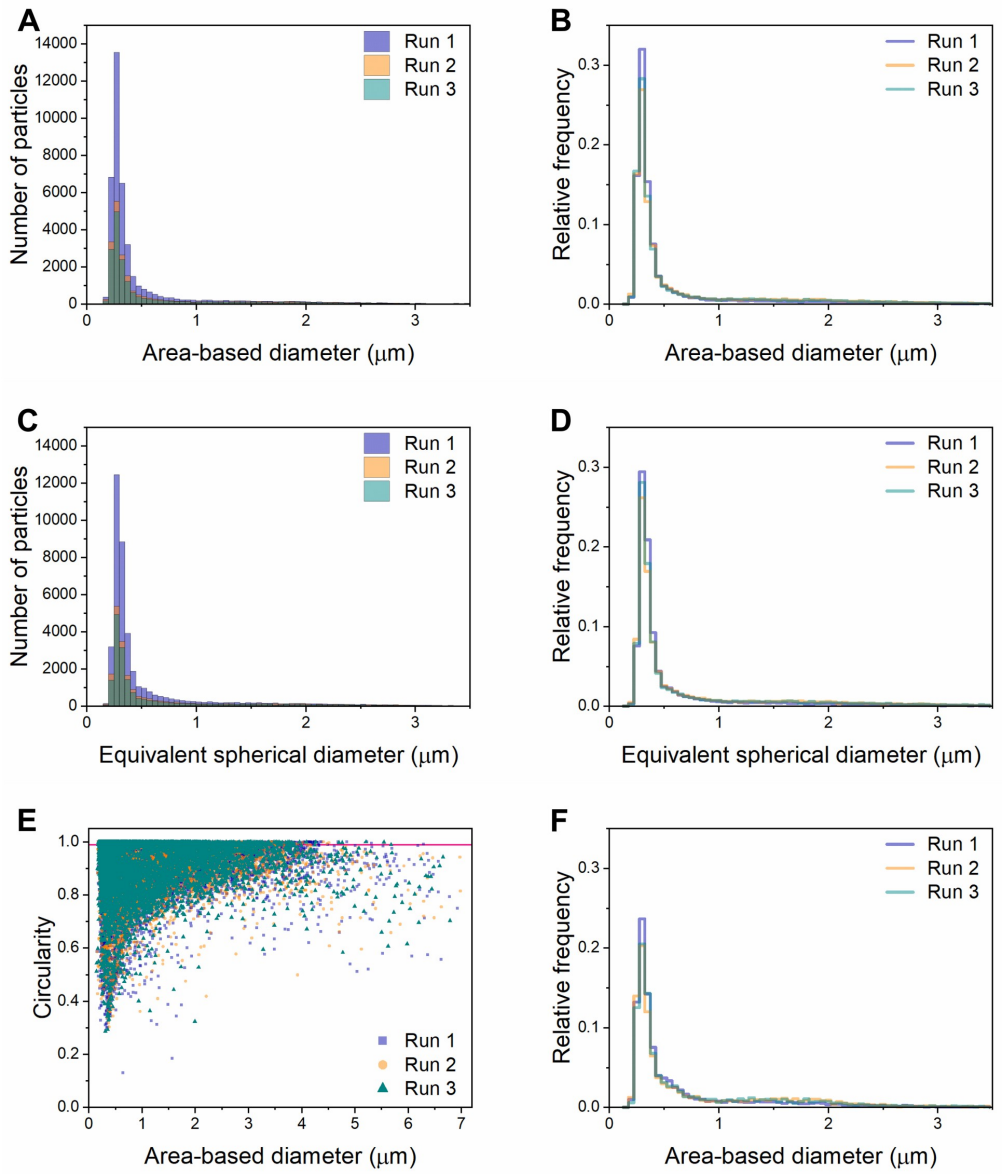
Size and shape characterization of I40 particles in water using the FlowCam Nano. Size distribution histograms as defined by area-based diameter (A and B) and equivalent spherical diameter (C and D) for triplicate runs of I40 in water. Panels A and C show the total number of particles, while panels B, D, and F are normalized to show relative frequency. (E) Scatter plot of circularity versus area-based diameter. Pink line represents a circularity value = 0.99. (F) Histogram showing the size distribution of particles with circularity > 0.99.

**Fig 7:**
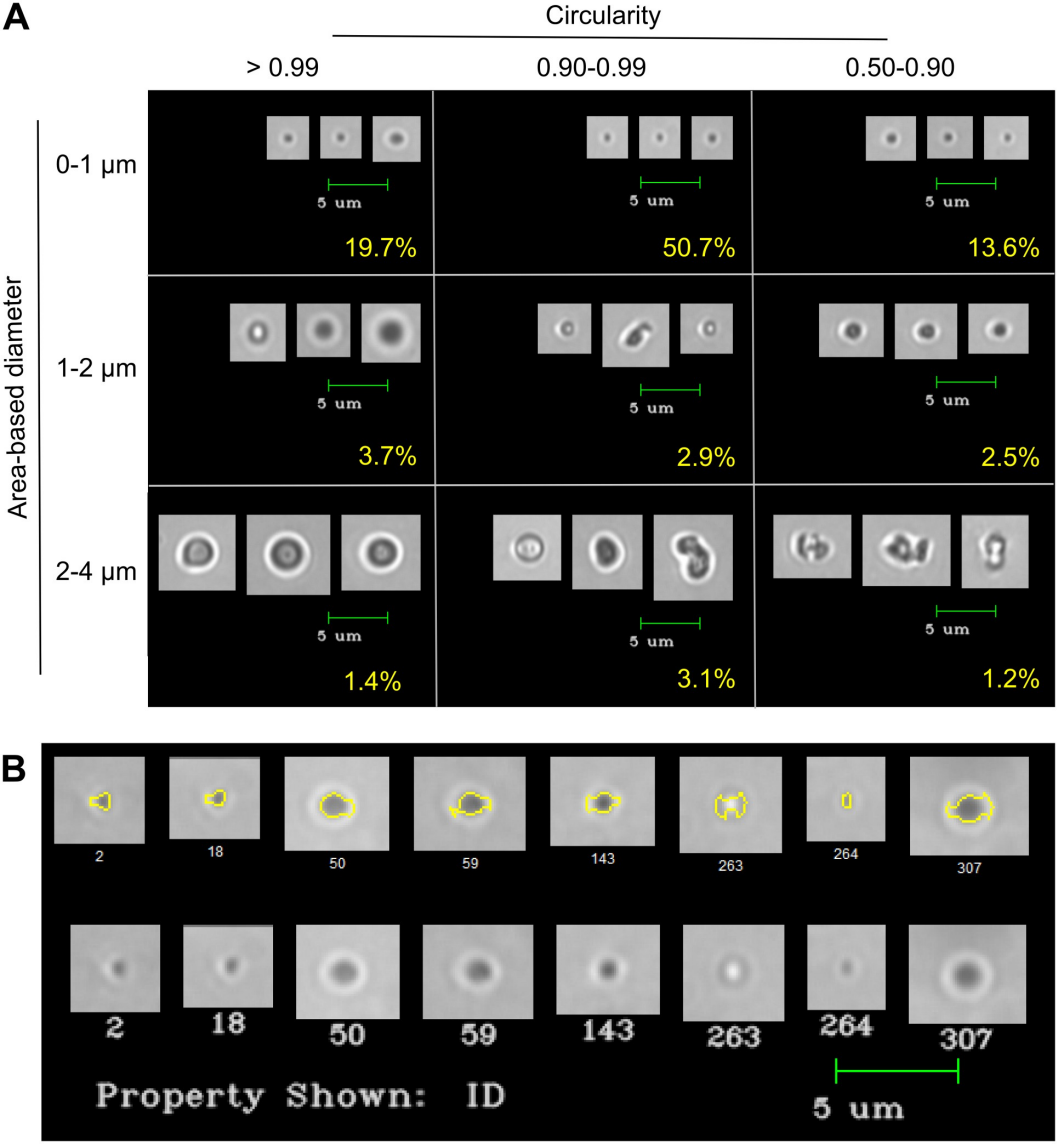
Representative images of I40 particles in water. (A) FlowCam Nano images were classified using filters corresponding to the indicated size and circularity bins. Classes were then sorted by edge gradient and the three images with the highest edge gradient from a single run were selected as representative of each class. Percentages of total particles classified into each filter bin are shown in yellow. (B) Top row: 0–1 μm particles classified as having low circularity (0.50–0.90) according to their particle edge traces (yellow). Bottom row: these same particles shown without the particle edge trace appear more circular to the human eye.

We also analyzed I40 samples on the FlowCam Nano, which uses oil immersion microscopy to image particles at higher magnification and thus provides substantially more information about particles with diameters 200 nm– 2 μm. In the experiments shown in Figs 6 and 7, the majority of particles were in the submicron range, with a mean ABD of 602 nm (664 nm ESD) and a median ABD of 306 nm (324 nm ESD). Therefore, the data collected on the FlowCam Nano is consistent with DLS sizing of sub-micron particles. For these data, high circularity was no longer a reliable metric for filtering single particles, as even the lower circularity bins contained single particles (Fig 7A). The classification of such particles as having low circularity may be at least partly explained by particle edge tracing errors (Fig 7B), which can be minimized by adjusting thresholding settings during data collection or in post-processing. However, overall, the average circularity of the Nano data was higher than that of the VS data (0.941 vs 0.847). This could be due to the ability of the Nano to capture images of single coacervate particles in the submicron range.

### Comparison of I40 particle analysis techniques

Overall, the above comparison of DLS, AFM, and flow imaging microscopy analysis of ELP coacervates underscores the fact that the “best” choice of technique will obviously depend on the needs of the researcher (Table 1). In particular, for analysis of ELPs and their assemblies, the most significant advantage of flow imaging microscopy is the large quantity of image-based data for morphological analysis facilitated by built-in automatic classification tools. However, the inability to observe nanoscale particles representing monomer and oligomer ELPs precludes full-scale investigation of coacervation or assembly processes using flow imaging alone.

**Table 1:** Comparison of techniques used in this analysis.

	Dynamic Light Scattering	Atomic Force Microscopy	FlowCam VS	FlowCam Nano
**Particle size measurement range**	1 nm–6 μm	<1 nm–30 μm	2–10+ μm	0.2–10+ μm
**Typical number of particles per measurement**	Distribution information only	1–300	10,000+	100,000+
**Shape analysis**	No	Yes	Yes	Yes
**Additional material information**	Aggregation, temperature dependence	Mechanical properties, nanostructure, microstructure	Microstructure, aggregation	
**Advantages**	Rapid, solution-based, size distribution information	High resolution, small amount of sample required	Rapid, solution-based, direct imaging for morphological analysis, high throughput permits robust statistics	
**Disadvantages**	No shape or morphology information, reproducibility issues for polymodal samples	Surface-based, low throughput, technical expertise required	Larger size range excludes soluble ELP monomers and oligomers, lacks temperature control	

When comparing results from different methods (e.g., for validation purposes), one must recognize that there are many different ways to derive even seemingly-straightforward particle characteristics such as particle diameter. DLS assigns particle diameter based on the size of a sphere with similar diffusion behavior. This calculation is not possible using image data from AFM or flow imaging microscopy, complicating efforts to directly validate one technique with another. Likewise, in both AFM and flow imaging, the accuracy of size calculations depends on image resolution and particle boundary assignments that may be influenced by both built-in software and user-determined options.

Finally, simply by virtue of the fact that flow imaging microscopy of ELP coacervates is new, artifacts and potential pitfalls associated with DLS and AFM are more easily anticipated and mitigated than those that have yet to be discovered for flow imaging analysis. For example, we did not investigate the influence of flow rate on particle shape or propensity for aggregation, but one might reasonably predict that there may be effects. Further careful study of these and similar materials is needed.

### Flow imaging assessment of I40 in different solvent conditions

The coacervation of ELPs such as I40 can be stimulated and tuned in various ways depending on the overall length and composition of the polymer. For example, I40 contains one Cys residue containing a thiol group that may be either reduced or oxidized depending on solvent conditions. In the absence of a reducing agent, such as in the preceding experiments performed in water, some fraction of I40 can be expected to behave as a longer polymer (i.e., have a lower *T*_t_) due to the formation of dimers via disulfide bonding. Our DLS and flow imaging analysis show that coacervation of I40 occurs to some extent at room temperature in water at our working concentration. As with other ELPs, I40 coacervation can also be triggered isothermally through the addition of salts such as NaCl. We asked whether flow imaging analysis of I40 coacervate solutions would reveal any differences in the particles formed under the influence of different solvent conditions. Specifically, we used flow imaging with the VS instrument to study the size and morphology of I40 particles formed in buffered high salt (3 M NaCl, frequently used in temperature transition cycling of ELPs) and typical reducing conditions for protein disulfides (5 mM DTT). Data were classified by size and circularity using the same bins as Fig 5. To allow reliable comparison of single coacervate particles between conditions, we created libraries of particles in the highest circularity bins (>0.99) for each condition from multiple runs (S1–S3 Files).

**Fig 8:**
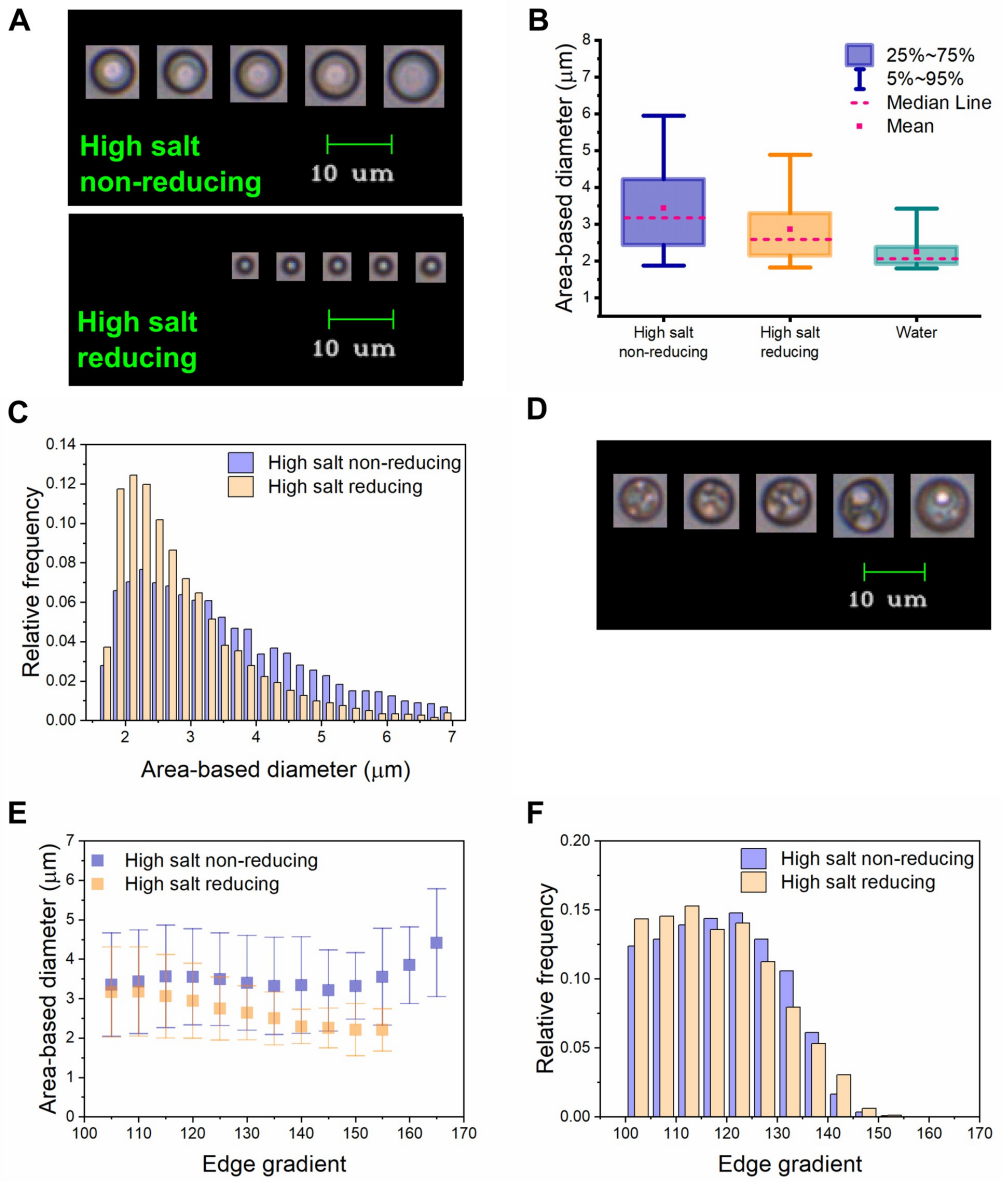
Comparison of I40 coacervates in different solvent conditions using flow imaging microscopy. (A) Libraries of particles with circularity values >0.99, representing single coacervates, were created for each solvent condition. Five images with the highest edge gradient values were selected from single runs as representative of each library. (B) Box-and-whisker plots showing the size distribution of I40 coacervates in different solvent conditions. The box represents the upper and lower quartiles and the whiskers represent the 5^th^ and 95^th^ percentiles. The mean and median are shown as a pink dot and a dashed line, respectively. Outliers are not shown. (C) Histogram of size distribution of I40 coacervates under high salt non-reducing and reducing conditions. (D) Representative images of internal structures visible within some larger coacervates. (E) Average diameter of I40 coacervates in high salt non-reducing and reducing conditions, binned by edge gradient values. Y-error bars represent ± standard deviation. (F) Histogram of edge gradient distribution of I40 coacervates under high salt non-reducing and reducing conditions.

In both non-reducing and reducing high salt conditions, particles were larger and more uniformly circular than in water at the same temperature (Fig 8A and 8C; compare the proportion of total images composed of single coacervates within each condition in Table 2). The larger size of I40 coacervates in high salt conditions relative to water is expected, as increasing ionic strength promotes coacervation in this range. Salt-driven coacervation would also be expected to promote the coalescence of oddly shaped, loosely aggregated I40 particles into larger, more circular assemblies. Images of the largest I40 particles allowed visualization of some internal organization, albeit at low resolution (Fig 8D). We hypothesize that these images show the coalescence of smaller coacervates into larger ones, consistent with the assembly route seen in DLS temperature trend experiments. Additionally, we hypothesize that the larger diameter observed in the absence of reducing agent can be attributed to cooperativity between coacervation and disulfide bond formation. As I40 polymers are driven to assemble via the hydrophobic effect, they are more likely to have the opportunity to form disulfide bonds; once such linkages are formed, reversibility of coacervation is decreased and the length-dependent effect described above is also in effect [40].

**Table 2:** Analysis of I40 coacervate size and shape by flow imaging microscopy.

Sample	Number of images (pooled runs)	Area-based diameter (μm)*	Equivalent spherical diameter (μm)*	Circularity*	Edge gradient*
**Water (VS)**	15,159 (3)	2.57 ± 0.83	3.09 ± 1.09	0.85 ± 0.11	112.8 ± 8.8
Single coacervates**	697	2.25 ± 0.55	2.52 ± 0.64	-	115.9 ± 10.3
**Water (Nano)**	80,365 (3)	0.60 ± 0.71	0.66 ± 0.79	0.94 ± 0.08	52.0 ± 10.7
Single coacervates**	20,028	0.68 ± 0.68	0.71 ± 0.70	-	51.1 ± 10.1
**High salt non-reducing**	18,437 (4)	3.49 ± 1.28	4.31 ± 1.63	0.99 ± 0.05	118.8 ± 11.0
Single coacervates**	16,920	3.43 ± 1.25	4.25 ± 1.60	-	119.0 ± 11.0
**High salt reducing**	13,397 (7)	3.13 ± 1.16	3.71 ± 1.47	0.92 ± 0.10	115.5 ± 10.7
Single coacervates**	5,156	2.86 ± 0.97	3.29 ± 1.15	-	118.1 ± 11.4

*Values are means ± standard deviation

**Defined as particles having a circularity value > 0.99

To ensure that the data sets used in comparing different solution conditions were of equivalent image quality, we used edge gradient as a metric. An edge gradient value between zero and 255 is automatically assigned to each particle image by the FlowCam software. This value is based on an edge detection algorithm that creates a mathematical gradient of the particle image intensity function. It represents the average intensity of the pixels comprising the border of the particle. Therefore, a higher edge gradient value may be correlated with a higher quality, more focused image. For data collected on the FlowCam VS, we applied an edge gradient filter with a cutoff value of 100. However, we were cautious not to apply any filters that would bias or invalidate comparisons between different conditions. For example, in Fig 8E, it can be seen that the average particle size in non-reducing conditions is larger than in reducing conditions across all edge gradient bins. The average edge gradient value and edge gradient distributions for both conditions were similar (Table 2, Fig 8F), suggesting that the images within the data sets being compared are of similar quality. However, if we had chosen to analyze only the top 300 most focused particles from each condition, the difference in the mean ABD values would have widened to 0.97 μm rather than 0.57 μm, i.e. a 70% inflation of the difference in average size. We must consider both the possibility that analyzing the top 300 most focused particles would provide more reliable information because the images are higher quality, and the possibility that doing so would generate biased information.

### Additional insights gained, best practices, and caveats

In addition to the particle edge trace concerns and edge gradient quality control measures mentioned above, what else should the researcher consider in experiments similar to ours? In the spirit of transparency and in the hopes of improving reproducibility in biomaterials characterization, we communicate some lessons learned and offer recommendations for future studies. We discuss concerns that we believe to be generally relevant to those studying protein and polymer assemblies, and specifically relevant to studies of ELPs.

#### Sample handling

In this study, three different independent preparations of I40 were used, each from a fresh transformation of *E*. *coli* with the same stock of sequenced plasmid DNA. We confirmed that these biological replicates behaved consistently throughout the purification process, SDS-PAGE, and flow imaging microscopy. Although we noticed variability in the appearance of freeze-dried ELP material (sometimes bulky, other times more fibrous), we did not notice any difference in solution or stimuli-responsive behavior once ELPs were re-suspended. As mentioned above, the formation of disulfide bonds negatively affects the reversibility of coacervation. For this reason, we used a freshly prepared re-suspension of I40 for each experiment, discarded samples after one day of storage at 4 °C, and were cognizant of the amount of time between re-suspension and analysis for each sample. While time was not a variable that we explored in this study, we believe that flow imaging microscopy would be an excellent tool to study the process of ELP coacervation over time.

#### Data quality

Upon coacervation, both the turbidity and the viscosity of ELP solutions are increased [41]. Increased solution turbidity may reduce edge gradient and result in variable and/or inaccurate particle sizing and counting [31,42]. Viscosity may influence the choices of flow rate and flow cell size used in an experiment, which in turn affect the optical resolution one can achieve. Flow issues such as the formation of bubbles and clogs during a run are exacerbated by the combination of viscous, coacervated, or highly concentrated ELP solutions and the narrow flow cells that permit the highest magnification.

**Fig 9:**
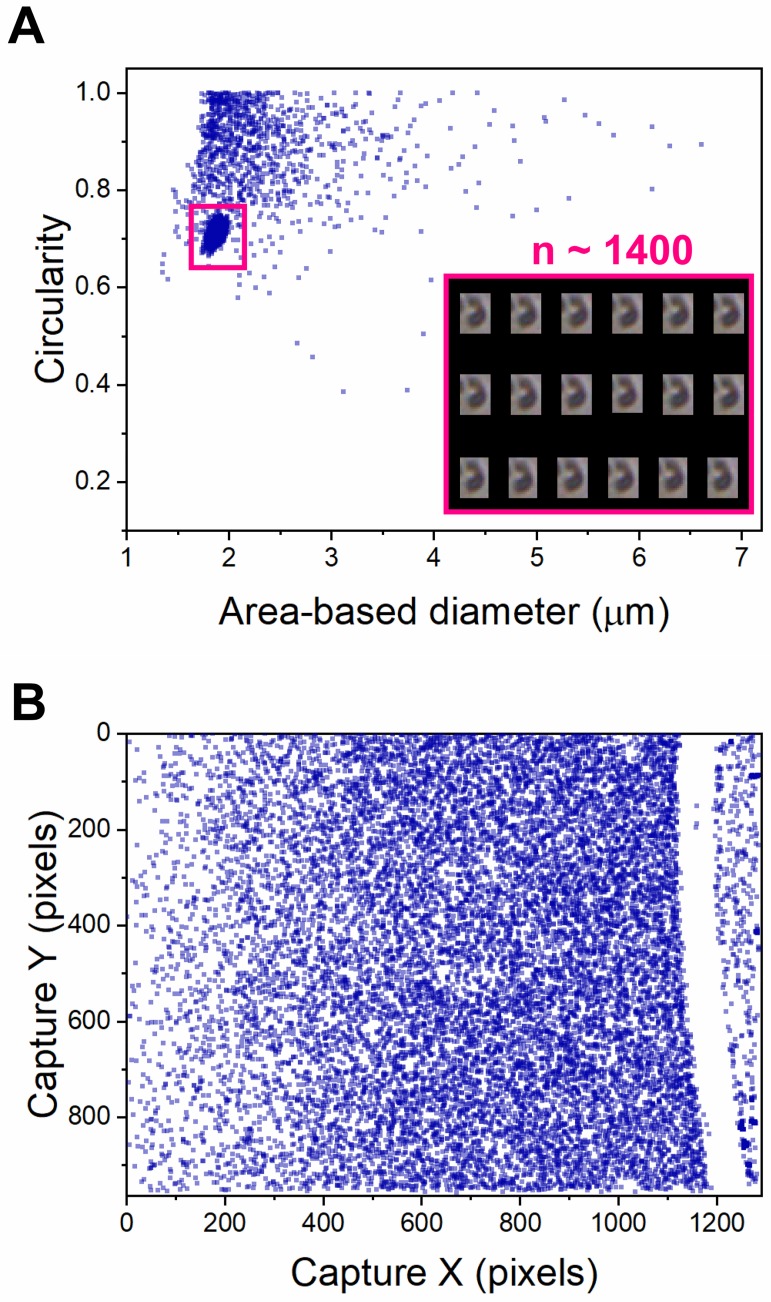
Scatter plots can provide useful information about the quality of flow imaging microscopy experiments. (A) Scatter plot of circularity versus area-based diameter for a single run of I40 in water. The distinct cluster of similar particle images (pink box) indicates that a single particle has been captured a very large number of times after getting stuck in the flow cell. (B) Scatter plot showing the position of each particle within the camera field of view for a single FlowCam run. The stripe without data points at x ≈ 1200 pixels indicates that something is preventing particles from entering the flow cell upstream of this x position.

Physisorption of ELPs to the flow cell during a run may result in repetitive image capture of the same particle (Fig 9A). This plot shows an unusual “hot spot” representing the same particle captured ~1400 times, comprising over half of the total particle images for this run. Repetitive images can be removed manually or through the use of filters in post-processing, but may not be immediately evident in particle characteristic data such as size or shape distributions. Additionally, aggregates or bubbles may lodge in the tubing before entering the flow cell, blocking normal flow. Fig 9B shows a scatter plot representing the camera’s two-dimensional field of view. In this representation, the direction of the flow is from top to bottom, and each point is the position of a particle captured during a single run. In this experiment, the flow was apparently physically disrupted by something (such as a bubble or large aggregate) before entering the flow cell, resulting in almost no particle images being captured at x ≈ 1200 pixels. While the particle images themselves resulting from this experiment were normal and usable, the capture X-Y plot served as a warning of potential aggregation or clogging problems in subsequent runs.

Given the importance of clean flow cells for data quality, we found it useful to designate several flow cells as “for protein use only.” In addition to preventing cross-contamination of other researchers’ samples with our proteins, this designation also allowed us to develop our own detergent-free cleaning procedures. This is important because the presence of some detergents may interfere with protein folding and assembly. For example, the anionic detergent SDS has been shown to influence the thermal behavior of ELPs [43,44].

#### Practicing responsible statistics

Researchers adopting flow imaging microscopy may find themselves suddenly confronted with the “problem” of having an enormous amount of data. One advantage of having a very large sample size is that the true distribution of a characteristic in a population is likely to be revealed in a well-constructed histogram. However, in the case of our data, histograms also revealed heavily skewed, non-normal size distributions, which is relevant in choosing statistical tests and interpreting descriptive statistics. The large number of images for each run also generated a very high degree of statistical power, resulting in differences being assigned statistical significance that likely have no practical significance. For example, we can refer to the above discussion of edge gradients in comparing single coacervates in high salt non-reducing and reducing conditions. The mean edge gradient values of these two libraries are 119.0 and 118.1, respectively (Table 2). Taken together with Fig 8F, we can say that there is probably no meaningful difference in the focus quality of these libraries. However, a two-sample t-test using the Welch correction for unequal variance applied to these same libraries assigns a *p* value = .000002 to the conclusion that these libraries are different. We have elected to report only descriptive statistics on our full particle libraries, as they are likely to be highly reliable based on the large number of particles measured (Table 2 and S2 Table). If inferential statistics are desired, we recommend generating a smaller sample from randomly selected data following a power analysis. For example, based on an expected effect size = 0.25 and alpha = 0.05, we selected 200 random data points from each of our different solvent libraries. When we performed a one-way analysis of variance (ANOVA) with Bonferroni post-hoc testing on these smaller data sets, the differences in diameter were determined to be statistically significant, while the differences in edge gradient were statistically non-significant (S2 Table).

## Conclusions

The primary goal of this study was to discover and describe the capabilities and limitations of flow imaging microscopy for ELP assemblies. Having demonstrated that ELP coacervates are amenable to evaluation by flow imaging microscopy, we were able to gain further insight into the nature of the populations of particles observed in DLS and AFM experiments. We found that, in some solution conditions, a considerable proportion of particles identified as micron-scale in diameter consist of aggregates of sub-micron-scale coacervates. We also found that for our I40 polymer, which has the ability to form a disulfide bond, the presence of a reducing agent affects the particle size and shape distributions.

In this study, we looked at a single (VPGIG)_40_ polymer under different solution conditions. Going forward, it will be interesting to investigate both the structure and dynamics of diverse ELP materials, including block co-polymers that form more programmable assemblies, polymers conjugated with different molecules for encapsulation or presentation, and fusion proteins with different functionalities. As flow imaging microscopy is currently used in assessing unwanted particles in pharmaceuticals, biopharmaceutical researchers are already well-equipped to adapt these methods for polymer microassemblies in drug delivery applications. Additionally, the adoption of flow imaging microscopy by the multidisciplinary biomaterials science community will not only create new knowledge of materials, but also accelerate the expansion of the technique itself, potentially through combination with other revolutionary tools such as super-resolution microscopy and materials data science.

## Supporting information

S1 Table Sequence information regarding the I40 POE-W construct. (XLSX)

S2 Table Descriptive statistics and one-way ANOVA of I40 coacervate libraries. (XLSX)

S3 Table List of FlowCam data sets used in this manuscript. (XLSX)

S1 Fig Raw image of SDS-PAGE of I40 purification. (TIF)

S2 Fig Raw height image from AFM analysis of I40 on mica. (TIF)

S1 File FlowCam data for library of I40 coacervates in water. (CSV)

S2 File FlowCam data for library of I40 coacervates in high salt non-reducing conditions. (CSV)

S3 File FlowCam data for library of I40 coacervates in high salt reducing conditions. (CSV)
