# Supplementary figures and images for: Flow imaging microscopy as a novel tool for high-throughput evaluation of elastin-like polymer coacervates
Source: PLoS One. 2019 May 9;14(5):e0216406. doi: 10.1371/journal.pone.0216406 (PMC6508725; doi:10.1371/journal.pone.0216406)

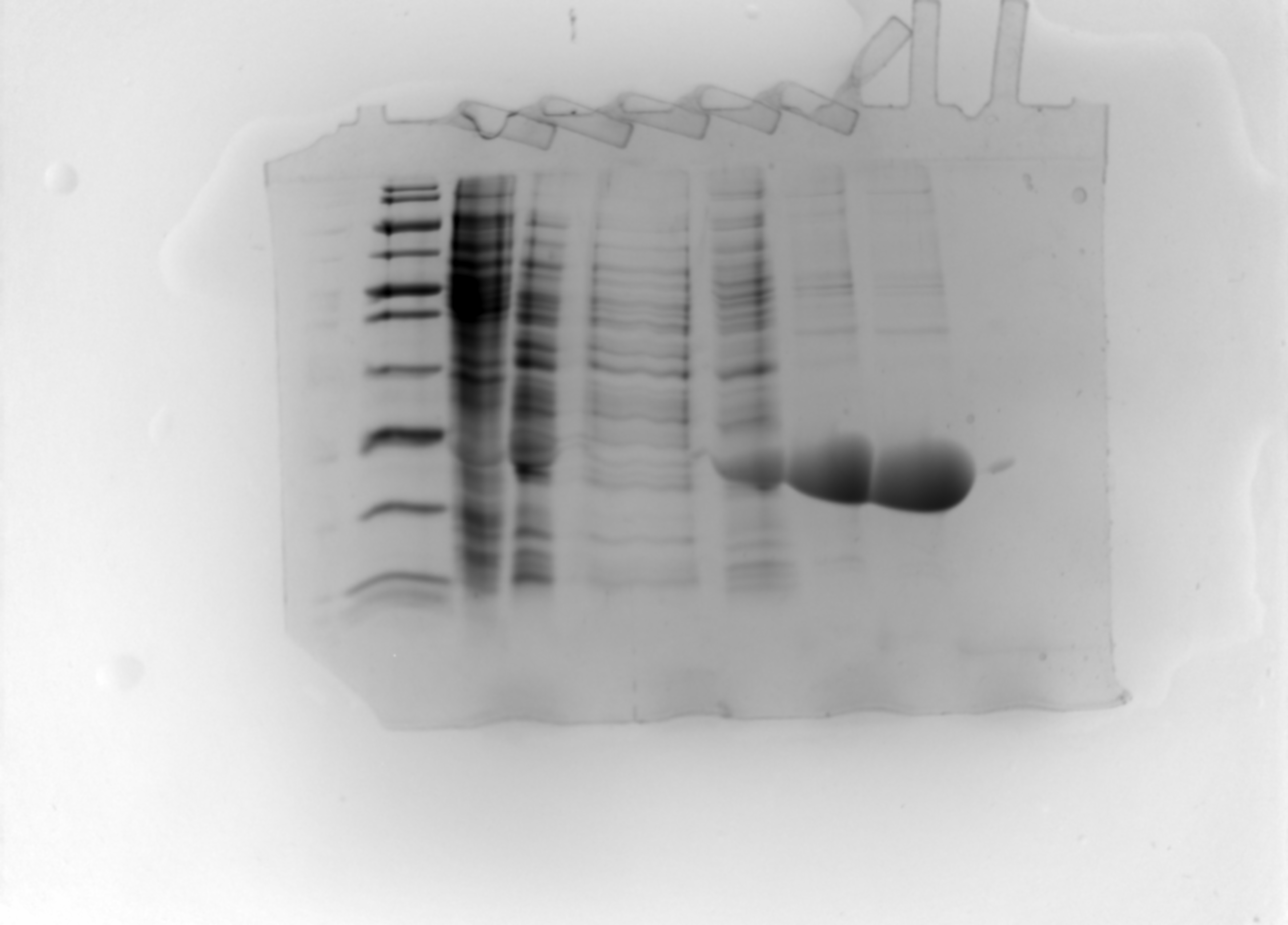

Supplement: S1 Fig — (TIF) [file pone.0216406.s004.tif]

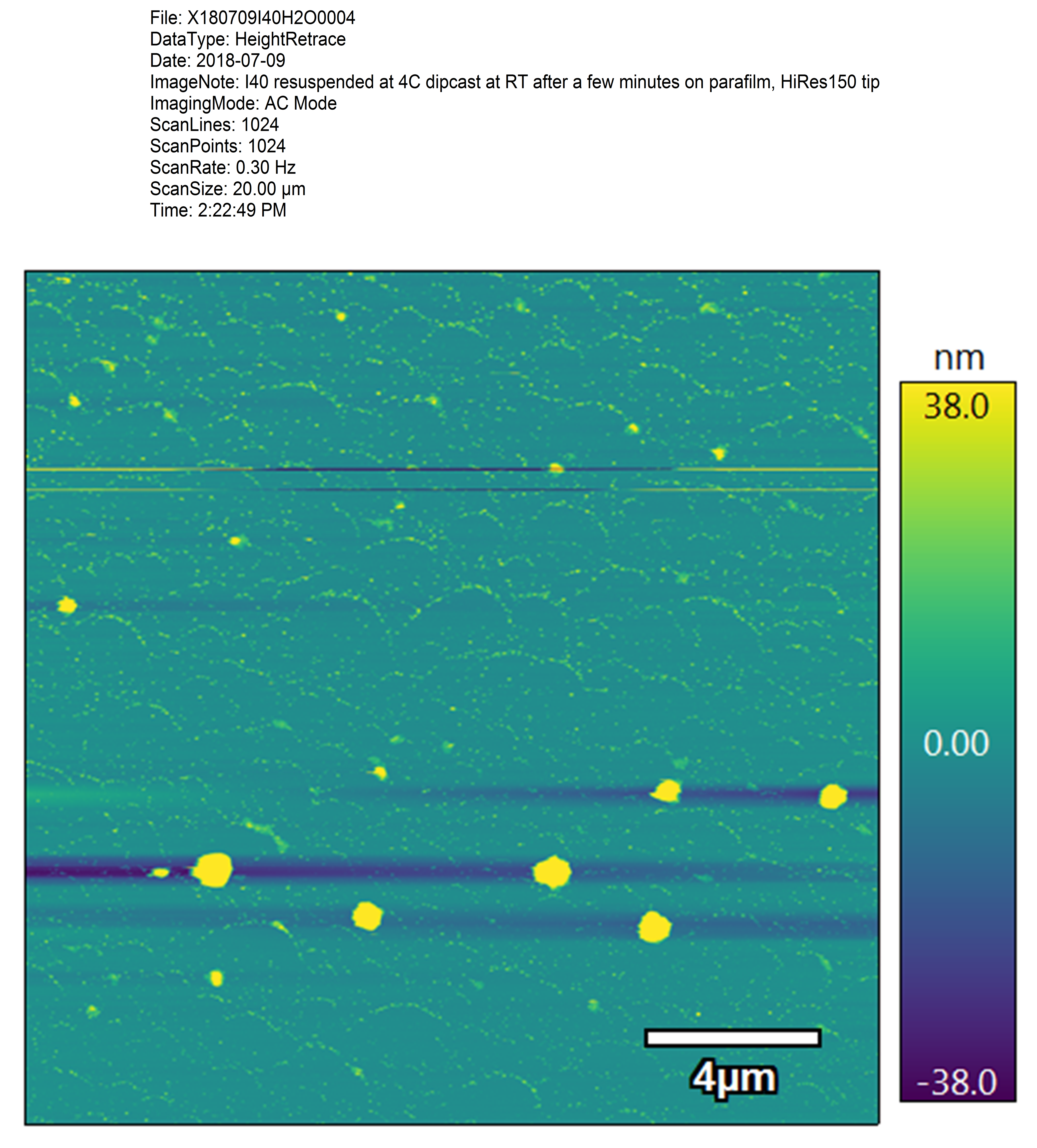

Supplement: S2 Fig — (TIF) [file pone.0216406.s005.tif]
